# Acupuncture for cancer-related hiccups: Protocol for a systematic review and meta-analysis of randomized controlled trials

**DOI:** 10.1097/MD.0000000000019973

**Published:** 2020-05-15

**Authors:** Rui Ma, Yan Li, Songjiang Liu, Wenhui Zhao

**Affiliations:** a Department of Otolaryngology, the First Affiliated Hospital of Heilongjiang University of Traditional Chinese Medicine; b First affiliated Hospital, Heilongjiang University of Chinese Medicine; c Harbin Medical University Cancer Hospital, Harbin, China.

**Keywords:** acupuncture, cancer-related hiccups, meta-analysis, protocol

## Abstract

**Background:**

Hiccups are involuntary contractions of the diaphragm and intercostal muscles, which lead to sudden contractions of the glottis. CAM, such as acupuncture is commonly used and stimulation of the vagus nerve and interference with phrenic nerve conduction are also used to treat hiccups. However, there is little evidence on the effectiveness of acupuncture for cancer-related hiccups. We will plan to conduct a systematic review and meta-analysis of RCTs to evaluate the current evidence on the effects of acupuncture for cancer-related hiccups.

**Method:**

The following databases will be searched: PubMed, the Cochrane Library, Wanfang Data, China National Knowledge Infrastructure (CNKI), SinoMed, VIP, Medline, Embase, and EI. Randomized controlled trials will be included to evaluate the effect and safety of acupuncture on cancer patients with hiccups. We will set standards for the curative effect on the basis of the standard of cure and improvement for clinical disease diagnoses. The risk of bias will be assessed by the Cochrane risk of bias tool. We will conduct a meta-analysis and sensitivity analysis, as well as a subgroup analysis if high heterogeneity is present, using Revman 5.3. We will use funnel plots to identify potential reporting biases. We will test asymmetry using Egger test. The Grading of Recommendations Assessment, Development, and Evaluation (GRADE) will be used to evaluate the quality of evidence.

**Results:**

This study will be to assess the effect and safety of acupuncture for cancer-related hiccups.

**Conclusions:**

This study will assess the effect of acupuncture for cancer-related hiccups and provide reliable evidence for the choice of treatments.

## 1 Introduction

### 1.1 Description of the condition

Hiccups are involuntary contractions of the diaphragm and intercostal muscles, which lead to sudden contractions of the glottis.^[1,2]^ The main symptoms of hiccups include continuous or intermittent short and frequent sounds caused by inverse gas in the throat.^[3]^ Intractable hiccups last longer than 48 hours.^[4]^ Cancer, radiotherapy, chemotherapy, and other cancer-related problems often cause hiccups.

The main reason for cancer-related hiccups is an increase in excitability of the phrenic nerve and vagus nerve. First and foremost, there are organic lesions, which are characterized as either central lesions or peripheral lesions.^[5]^ Hiccups caused by a central lesion involve the compression of hiccup reflex centers by intracranial and cervical tumors. Hiccups caused by peripheral lesions involve a tumor that metastasizes to the upper diaphragm or diaphragm (invaded by malignant tumors) or subdiaphragmatic or mediastinal carcinomas, such as gastric carcinoma, lung carcinoma, or alimentary canal carcinoma. Second, cancer-related treatments, such as chemotherapy and radiotherapy, lead to hiccups.^[5]^ Third, electrolyte disturbances and acid-base imbalances cause hiccups in patients with tumors.^[5]^

Currently, treatment options for cancer-related hiccups include medications, surgical treatment, complementary, and alternative medicine (CAM), stimulation of the vagus nerve and interference with phrenic nerve conduction. The medications that are often used include dopamine receptor antagonists, such as metoclopramide^[6]^; GABA-B receptor agonists^[7]^; antipsychotics, such as chlorpromazine^[8]^ and haloperidol; antiepileptic drugs, such as sodium valorous, carpentry,^[9]^ and carbamazepine^[10]^; calcium antagonists, such as nifedipine^[11]^ and nimodipine^[12]^; acid suppressants, such as omeprazole; central excitatory drugs, such as methylphenidate; and narcotic drugs, such as lidocaine and propofol. Surgical treatments, including unilateral and bilateral phrenic nerve block, are often used.^[13]^ CAM, such as acupuncture and moxibustion therapy, which includes milliacupuncture, a combination of scalp acupuncture and body acupuncture, electroacupuncture, moxibustion, and ear acupuncture, is used.^[14]^ Last, stimulation of the vagus nerve and interference with phrenic nerve conduction are also used to treat hiccups.^[5]^

### 1.2 Description of the intervention

Acupuncture has been used for over 2000 years to treat various diseases in China. It was first described in the Yellow Emperors Classic of Internal Medicine.^[15]^ Acupuncture is defined by the insertion of filiform needles into the skin at precise locations (acupuncture points) or other specific areas^[16]^ and the use of twiddle and lifting techniques to treat diseases. Acupressure is known as the noninvasive form of acupuncture. Acupuncture is simple, convenient, effective, and inexpensive, and it has few side effects^[17]^; it is gaining attention as a treatment for hiccups.^[18]^ Acupuncture is one of the major treatments used in traditional Chinese medicine and is widely used for the supportive and palliative care of patients with cancer.^[19]^

In 2012, systematic reviews (SRs) were conducted to summarize the evidence on the use of acupuncture for the management of cancer-related hiccups.^[20]^ There is little evidence on the effectiveness of acupuncture for cancer-related hiccups, which was summarized in this systematic review. The total number of RCTs included in the analysis was low, and the risk of bias for the RCTs was high. Therefore, it is of great importance to perform systematic reviews and meta-analyses of the randomized controlled trials (RCTs) on the effects of acupuncture in treating cancer-related hiccups. In this study, we plan to conduct a systematic review and meta-analysis of RCTs to evaluate the current evidence on the effects of acupuncture for cancer-related hiccups.

## 2 Methods

### 2.1 Eligibility criteria

#### 2.1.1 Types of studies

Randomized controlled trials will be included to evaluate the effect and safety of acupuncture in cancer patients with hiccups.

#### 2.1.2 Types of participants

Participants diagnosed with acute, persistent, or intractable hiccups for cancer will be included. The participants will include patients who have hiccups as a result of various cancers, including central lesion cancer and peripheral lesion cancer, and cancer-related treatments, including chemotherapy, radiotherapy, other medicines administered for cancer, and electrolyte disturbance and acid-base imbalance treatments. Patients with series of hiccups caused by an issue unrelated to cancer will be excluded.

#### 2.1.3 Type of interventions

The only type of intervention included for the treatment of hiccups in cancer patients is acupuncture. Acupuncture combined with other therapeutic methods to treat hiccups related to cancer will be excluded. All randomized controlled trials (RCTs) using either traditional or contemporary acupuncture will be included. Traditional acupuncture is defined by the insertion of needles in classic meridian points. Regardless of the source of electrical stimulation or fine needle, contemporary acupuncture is defined by the insertion of needles in nonmeridian or trigger points. Treatment without needling will be excluded, such as laser acupuncture, acupressure, auricular acupuncture with a specific pressure device, pharmacoacupuncture, tap-pricking, point injection, or moxibustion. There will be no restrictions on the duration and duration of treatment.

#### 2.1.4 Types of comparisons

Types of comparisons will include medication, no treatment, placebo acupuncture, and sham acupuncture. For example, chlorpromazine, sodium valorous, carbamazepine, nifedipine, omeprazole, lidocaine, and other medicinal treatments for hiccups related to cancer will be included. Valsalva movement, drinking a large amount of water, and other physiotherapeutic treatments for hiccups related to cancer will be included.

#### 2.1.5 Types of outcome measures

We set standards for the curative effect on the basis of the standard of cure and improvement for clinical disease diagnoses. When the hiccups stop after treatment and their associated symptoms resolve, the hiccups are considered to have been cured. The relief of, shortened duration of, or increased intervals between hiccups after treatments indicate improvement in patients. When the hiccup duration and hiccup attacks do not exhibit obvious changes, ineffectiveness of the treatment is noted.

#### 2.1.6 Types of secondary measures

The secondary outcomes include adverse events related to the interventions.

### 2.2 Search strategy and identification of study

The following databases will be searched: PubMed, the Cochrane Library, Wanfang Data, China National Knowledge Infrastructure (CNKI), SinoMed, Chinese Science and Technology Periodical Database (VIP), Medline, Embase and EI. The search will be conducted in Chinese and English with the following terms: (Acupunct OR body acupuncture OR scalp acupuncture OR auricular acupuncture OR Electroacupuncture OR fire needling OR elongated needle OR intradermal needling OR acupuncture OR auricular acupuncture OR scalp acupuncture OR needle OR acupuncture point OR acupoint OR acupuncture treatment OR acupuncture therapy) AND (hiccup OR hiccough OR hiccups OR hiccupping OR singultus OR intractable hiccup OR intractable hiccups) AND (cancer OR tumor OR carcinoma). Table 1 shows the search strategy used for PubMed.

**Table 1:**
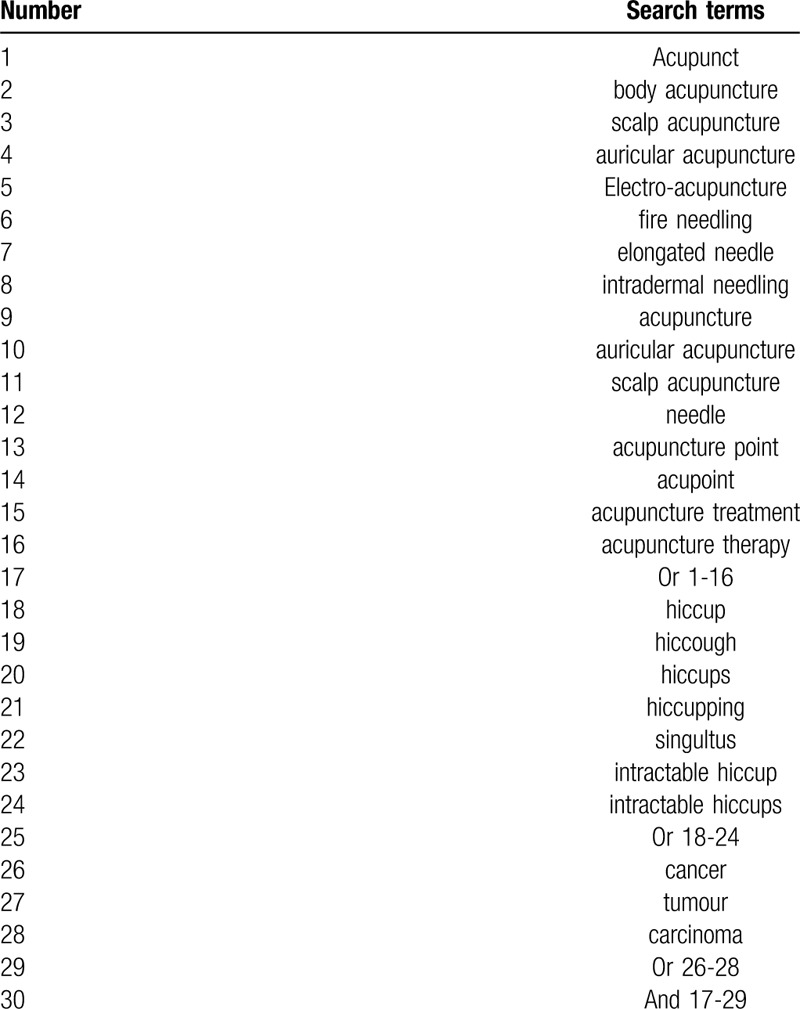
Search strategy for the PubMed database.

### 2.3 Data collection and analysis

#### 2.3.1 Selection of studies

**Figure 1:**
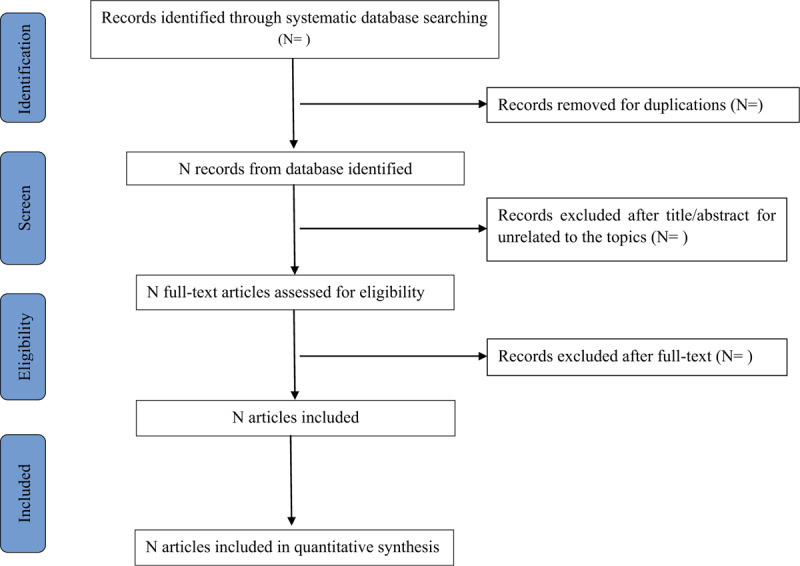
Flow chart of study selection.

The titles and abstracts will be selected and identified by 2 independent reviewers (MR and LY). The full text will be assessed. If there are any disagreements, they will be discussed and resolved by an experienced reviewer (L-SJ). All articles will be stored in a separate database. The 2 reviewers will independently assess all eligible studies according to the inclusion criteria. The Preferred Reporting Items for Systematic Reviews and Meta-Analyses (PRISMA) flow chart will be used to report the reasons for excluding and including eligible studies. The study selection procedure will be shown in a PRISMA flow chart (Fig. 1).

#### 2.3.2 Data extraction and management

All data from the studies will be independently extracted by 2 reviewers (MR and LY) according to the criteria previously described. We will create a standardized data extraction form to record the extracted data. The data extraction form will include the following items: general information, author, trial characteristics (RCT), year of publication, participants, inclusion and exclusion criteria, acupuncture intervention, control intervention, and outcomes measures. If there are any disagreements, we will discuss them with a third experienced reviewer (L-SJ) to resolve them. When data is unavailable, the corresponding authors will be contacted to obtain the missing data.

#### 2.3.3 Assessment of risk of bias in included studies

The risk of bias for all included studies will be assessed by 2 independent authors (MR and LY) according to the Cochrane Handbook for Systematics Reviews of Interventions. Any disagreements will be settled by a third experienced reviewer (L-SJ). The following aspects will be assessed for risk of bias: selection, performance, detection, attrition, reporting, and other factors. All included studies will be classified into 3 categories: high risk, low risk, and unclear level of risk.

#### 2.3.4 Measure of treatment effect

For dichotomous outcomes, we will calculate risk ratios (RRs) with 95% CIs. We will calculate mean differences (MDs) with 95% CIs for the continuous outcomes. If different scales are used to measure outcomes, we will use standard mean differences (SMDs) with 95% CIs.

#### 2.3.5 Unit of analysis issues

The unit of analysis will be all individuals participating in the trials.

#### 2.3.6 Missing data

When there are missing data, we will call or email the original authors of the articles to obtain the missing data. However, if missing data cannot be obtained, we will only use the data available.

#### 2.3.7 Assessment of heterogeneity

We will assess the statistical heterogeneity using *χ*^2^ tests (*P* = .1). We will check the *I*^2^ statistic to quantify the level of inconsistency. When *I*^2^ < 50%, the trials can be considered to represent low heterogeneity. When *P* < .1 or *I*^2^ > 50%, the trials can be considered to represent high heterogeneity. We will perform subgroup analyses if high heterogeneity exists.

#### 2.3.8 Assessment of reporting biases

We will use funnel plots to identify potential reporting biases. We will test asymmetry using Egger test.

#### 2.3.9 Data synthesis

Data synthesis will be performed by Review Manager 5.3. We will use a fixed effects model if there is no statistical heterogeneity. We will use a random effects model if high heterogeneity is found.

#### 2.3.10 Subgroup analysis

We will perform subgroup analyses if there is high heterogeneity in the included studies. Subgroup analyses will be conducted according to the age, sex, cause of hiccups, type of hiccups, type of acupuncture (acupuncture, electroacupuncture, etc.), type of control group (placebo, sham acupuncture, no treatment or medication), different acupoints of the electroacupuncture or course of treatment.

#### 2.3.11 Sensitivity analysis

To evaluate the robustness of the pooled results, we will perform a sensitivity analysis to assess the impact of the trials with a high risk of bias. We will compare the findings to determine whether lower quality studies should be excluded based on the size of the sample, the strength of the evidence and its impact on the overall scale of effectiveness.

#### 2.3.12 Grading the quality of evidence

The Grading of Recommendations Assessment, Development and Evaluation (GRADE) will be used to evaluate the quality of evidence. The studies will be assigned 1 of 4 possible ratings: very low, low, moderate, or high.

## 3 Discussion

The effective treatment of acupuncture on cancer-related hiccups is significant. We will summarize the available evidence for acupuncture on cancer-related hiccups, and evaluate the effectiveness and the adverse effects of these treatments on cancer-related hiccups. Our findings may assist clinicians make clinical decisions and promising way for treatment of cancer-related hiccups.

## 4 Ethics and dissemination

The review will not need ethical approval because no issues of participant privacy exist. The results of this systematic review will provide evidence about the effect and safety of acupuncture for hiccups. The results will be disseminated through peer review.

## Author contributions

**Conceptualization:** Rui Ma, Yan Li, Songjiang Liu, Wenhui Zhao.

**Data curation:** Rui Ma, Yan Li.

**Funding acquisition:** Yan Li.

**Methodology:** Rui Ma.

**Software:** Rui Ma, Yan Li.

**Supervision:** Songjiang Liu, Wenhui Zhao.

**Writing – original draft:** Rui Ma, Yan Li.

**Writing – review & editing:** Songjiang Liu, Wenhui Zhao.
